# A mouse model of brittle cornea syndrome caused by mutation in *Zfp469*

**DOI:** 10.1242/dmm.049175

**Published:** 2021-09-22

**Authors:** Chloe M. Stanton, Amy S. Findlay, Camilla Drake, Mohammad Z. Mustafa, Philippe Gautier, Lisa McKie, Ian J. Jackson, Veronique Vitart

**Affiliations:** MRC Human Genetics Unit, Institute of Genetics and Cancer, The University of Edinburgh, Western General Hospital, Crewe Road, Edinburgh EH4 2XU, UK

**Keywords:** Brittle cornea, Keratocyte, Zfp469, ZNF469, Collagen

## Abstract

Brittle cornea syndrome (BCS) is a rare recessive condition characterised by extreme thinning of the cornea and sclera. BCS results from loss-of-function mutations in the poorly understood genes *ZNF469* or *PRDM5*. In order to determine the function of ZNF469 and to elucidate pathogenic mechanisms, we used genome editing to recapitulate a human *ZNF469* BCS mutation in the orthologous mouse gene *Zfp469*. Ophthalmic phenotyping showed that homozygous Zfp469 mutation causes significant central and peripheral corneal thinning arising from reduced stromal thickness. Expression of key components of the corneal stroma in primary keratocytes from *Zfp469*^BCS/BCS^ mice is affected, including decreased *Col1a1* and *Col1a2* expression. This alters the collagen type I/collagen type V ratio and results in collagen fibrils with smaller diameter and increased fibril density in homozygous mutant corneas, correlating with decreased biomechanical strength in the cornea. Cell-derived matrices generated by primary keratocytes show reduced deposition of collagen type I, offering an *in vitro* model for stromal dysfunction. Work remains to determine whether modulating ZNF469 activity will have therapeutic benefit in BCS or in conditions such as keratoconus in which the cornea thins progressively.

This article has an associated First Person interview with the first author of the paper.

## Abstract (precis)

**Summary:** A mouse model of brittle cornea syndrome was created to elucidate molecular mechanisms underlying the pathology of this rare connective tissue disorder in which extremely thin corneas rupture, causing irreversible blindness.

## INTRODUCTION

Brittle cornea syndrome (BCS; OMIM 229200; OMIM 614170) is a rare autosomal recessive disorder that is characterised by extreme thinning of the cornea and sclera. Visual impairment may initially be a result of myopia and progressive keratoconus or keratoglobus but, as the name suggests, the thin and fragile corneas of affected individuals are prone to rupture leading to irreversible blindness (Al-Hussain et al., 2004). Also classified as a subtype of Ehlers–Danlos syndrome (EDS type VIB), this devastating condition often leads to general connective tissue dysfunction with skin hyperelasticity, joint hyperflexibility and, in approximately one-third of cases, hearing impairment (Malfait et al., 2017).

BCS results from biallelic loss-of-function (LOF) mutations in *ZNF469* or *PRDM5* (Abu et al., 2008; Burkitt Wright et al., 2011). Mutations in these genes appear to cause an indistinguishable disorder, suggesting that they contribute to the same biological pathways. PRDM5 (PR/SET Domain 5), a widely expressed transcription factor that modulates development and maintenance (Duan et al., 2007; Meani et al., 2009; Porter et al., 2015) is known to play an important role in extracellular matrix (ECM) production by several tissues, including skin fibroblasts (Porter et al., 2015) and bone (Galli et al., 2012). The role played by *ZNF469* in the healthy cornea and in BCS is less clear as the function of the very large protein encoded by this gene is poorly characterised. Since mutations in *ZNF469* were first reported to cause BCS (Abu et al., 2008), 30 pathogenic compound heterozygous or homozygous mutations have been identified in the single coding exon of *ZNF469* spanning 13 kb on chromosome 16q24.2 (Abu et al., 2008; Al-Owain et al., 2012; Christensen et al., 2010; Dhooge et al., 2021; Khan et al., 2012, 2010; Menzel-Severing et al., 2019; Micheal et al., 2019; Ramappa et al., 2014; Rohrbach et al., 2013; Rolvien et al., 2020; Skalicka et al., 2020). The majority of these mutations result in premature stop codons that are hypothesised to lead to the production of truncated and non-functional protein (Rohrbach et al., 2013), and are thus considered as LOF mutations.

As *ZNF469* encodes a C2H2 zinc-finger protein, it is assumed to function as a transcriptional regulator for the synthesis or assembly of collagen fibrils (Abu et al., 2008). Studies in dermal fibroblasts showed that mutations in ZNF469 or PRDM5 affect the expression of fibrillar collagen genes and the deposition of collagen into the ECM (Burkitt Wright et al., 2011). In humans, 90% of corneal thickness [normal range 450-700 µm (Vitart et al., 2010)] is contributed by the stroma (Alberto and Garello, 2013), a collagen-rich ECM deposited by resident keratocytes. The precise organisation of collagen fibrils in the stroma is crucial to cornea function, combining mechanical strength and almost perfect transmission of visible light to provide approximately two-thirds of the refractive power of the human eye. Stromal disorganisation and thinning are seen in many corneal disorders, including keratoconus [affecting 1/2000 individuals in the UK (Pearson et al., 2000)], but the reduction in central corneal thickness (CCT) observed in BCS is extreme (<400 µm).

The underlying biological processes that lead to corneal thinning in BCS are not well understood, but may reflect pathological extremes of the processes determining corneal thickness in healthy eyes. This is supported by heterozygous carriers of *ZNF469* and *PRDM5* mutations, who have mildly reduced CCT relative to homozygotes (Burkitt Wright et al., 2011), and by the association of variants in putative regulatory elements of *ZNF469* with reduced CCT in the general population (Iglesias et al., 2018; Lu et al., 2010, 2013). Dysregulation of collagen synthesis or assembly by keratocytes is known to have a strong effect on corneal thickness (Dimasi et al., 2010; Evereklioglu et al., 2002; Segev et al., 2006; Sun et al., 2011). However, the role that ZNF469 plays in keratocyte cells remains undetermined. To address this, we generated the first mouse model of BCS caused by mutation in the orthologous mouse gene *Zfp469* to elucidate the mechanisms by which stromal thickness is controlled in health and disease.

## RESULTS

### Zfp469 is the mouse orthologue of human ZNF469 and can be modified to recapitulate human BCS mutations

**Fig. 1.**
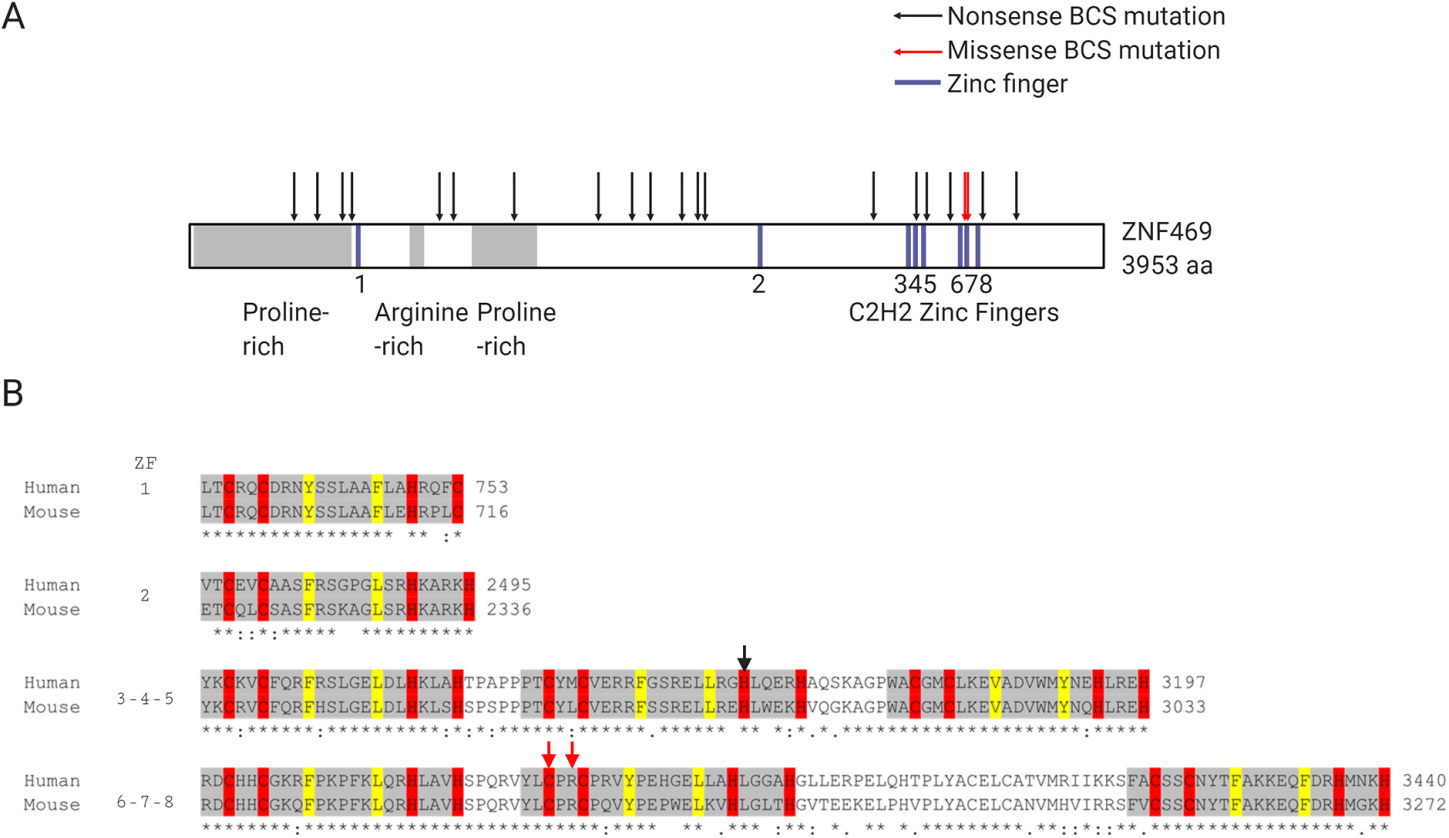
**Pathogenic mutations in ZNF469 are distributed throughout the protein and result in premature termination codons or disruption of conserved residues in C2H2 zinc-finger domains.** (A) Schematic representation of the protein structure of ZNF469, showing the position of previously reported pathogenic variants in relation to eight C2H2 zinc-finger domains and regions of compositional bias. Nonsense or frameshift variants in C2H2 ZF domains resulting in a premature stop are indicated by black arrows; missense mutations are indicated by red arrows. (B) Sequences of the eight C2H2 zinc-finger (ZF) domains in the mouse (Zfp469, NP_001354553.1) and human (ZNF469, NP_001354553.1) proteins were aligned using Clustal Omega. Fully conserved residues are indicated by ‘*’; ‘:’ indicates conservation between groups of amino acids with strongly similar properties; and ‘.’ indicates conservation between groups of amino acids with weakly similar properties. Residues shown in red are the paired cysteines (C) and histidines (H), which bind the zinc ion. Residues in yellow are structurally important hydrophobic amino acids.

We performed a literature review to compile a comprehensive list of mutations in *ZNF469* that have been identified in BCS families to date (March 2021), and mapped each mutation to human *ZNF469* transcript NM_001367624.2 (Table 1). Of the 30 genetic variants that have been reported as homozygous or compound heterozygous mutations in BCS cases, 27 (90%) result in either nonsense or frameshift mutations in ZNF469, which are thus predicted to result in the production of a truncated protein. These mutations occur throughout the *ZNF469* coding sequence (NP_001354553.1; Fig. 1A), and, with the exception of one large deletion encompassing the gene (Ramappa et al., 2014), share common consequences of truncating the protein or, in the case of two missense mutations, modifying key residues involved in the coordination of zinc in the sixth C2H2 zinc-finger domain. Fewer LOF mutations than expected have been seen in human populations {probability of being LOF intolerant (pLI)=0.72 [observed/expected=0.2 (0.12-0.37, 90% c.i.)] in gnomAD (Lek et al., 2016)}. Only four of the BCS mutations reported in affected individuals, p.Gln2149Serfs*51, p.Gln2149Alafs*42, p.Arg3442Glyfs*59 and p.Pro3584Glnfs*136, have been identified in a small number of heterozygous individuals in gnomAD v3.1 (Table 1).

**Table 1.**
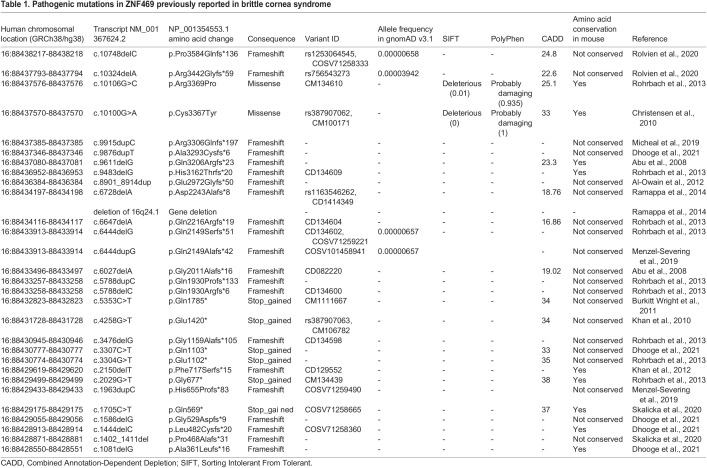
Pathogenic mutations in ZNF469 previously reported in brittle cornea syndrome

The orthologous gene in mouse, *Zfp469* or *Gm22*, is located on chromosome 8 (NM_001362883). Clustal Omega (Sievers et al., 2011) was used to align orthologous protein sequences from mouse (Zfp469, NP_001354553.1) and human (ZNF469, NP_001354553.1), with 47% amino acid identity across the full-length of each protein (3953 amino acids in human, and 3765 amino acids in mouse). The eight predicted C2H2 zinc-finger domains [positions obtained from the SMART database (Letunic et al., 2021)] show a much higher degree of conservation (Fig. 1B), consistent with zinc-finger domains having an important functional role. Of the human BCS mutations, only some affected conserved amino acids (Table 1). One of these (human p.Gly677*, mouse p.Gly634) was selected as the target for genome editing using CRISPR/Cas9n to create a mouse model of BCS.

### Gene editing *Zfp469* to generate *Zfp469*^BCS^ mice

**Fig. 2.**
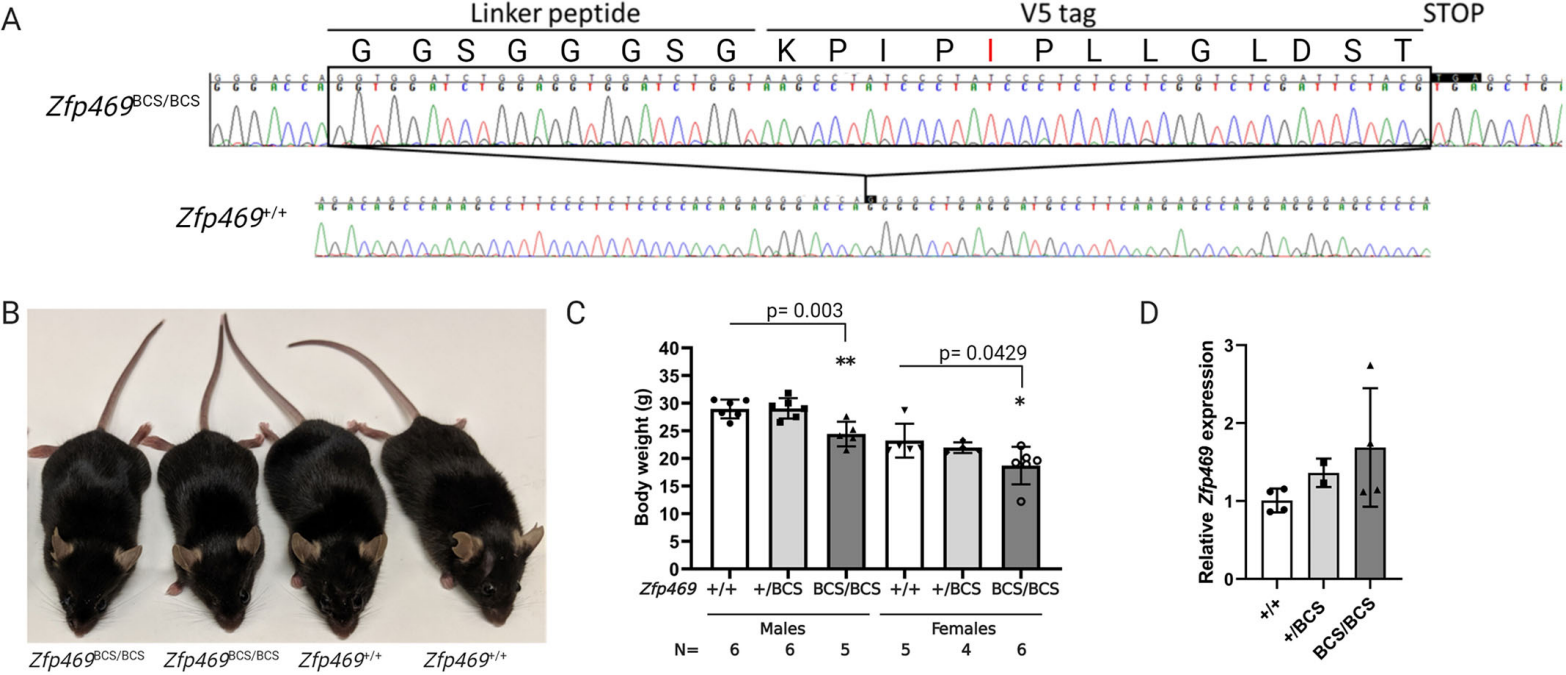
**Genome editing *Zfp469* to create a mouse model of brittle cornea syndrome (BCS).** (A) CRISPR/Cas9 genome editing was used to insert an in-frame V5 tag and premature stop codon into Zfp469 at p.Gly634, the conserved residue equivalent to the human mutation p.Gly677*. Sequencing chromatograms from a homozygous *Zfp469*^BCS/BCS^ mouse compared to a wild-type littermate indicating the position and sequence of the insertion. The V5 tag contained a spontaneously arising A>T mutation, resulting in the amino acid change Asn (N)>Ile (I) at position 5 in the V5 tag. (B) A representative image of two female *Zfp469*^BCS/BCS^ and two female *Zfp469*^+/+^ littermate mice at 3 months of age. *Zfp469*^BCS/BCS^ mice showed no gross phenotypic abnormalities but were smaller than *Zfp469*^+/+^ sex-matched littermates. (C) Bodyweight of *Zfp469*^BCS/BSC^ was significantly reduced relative to *Zfp469*^+/+^ age- and sex-matched animals, as determined by one-way ANOVA with Dunnett's multiple comparison test (*P<0.05, **P<0.01). Data are mean±s.d. (D) qPCR analysis revealed no significant difference in the relative expression of *Zfp469* in keratocytes isolated from the corneas of four wild-type, two heterozygous and four homozygous mice aged 3 months. Data are mean±s.d. with the average of assays performed in duplicate from each sample shown by data points.

To elucidate the role of Zfp469 in mice, genome editing was performed to recapitulate a human BCS mutation by creating a premature stop codon early in *Zfp469* prior to the C2H2 zinc-finger domains. Three pairs of sgRNA (Table S1) designed to target sequence encoding Zpf469 p.Gly634 were cloned into pX458 (pSpCas9(BB)-2A-GFP) (Ran et al., 2013) and tested for cleavage efficiency in mouse embryonic fibroblasts (MEFs). The pair of sgRNAs with the highest cleavage efficiency (gRNA1 and gRNA3) was subsequently *in vitro* transcribed, and purified RNA was injected, along with SpCas9n mRNA and the single-stranded donor oligonucleotide (ssODN) repair template, into C57Bl/6J mouse zygotes. Successful genome editing and repair inserted an in-frame V5 tag, and generated a premature stop codon to truncate Zfp469 (Fig. 2A). Homozygous mutant mice were significantly smaller than their sex-matched littermate controls at 3 months of age (Fig. 2B,C), weighing 15-20% less than wild-type mice (males, *P*=0.003; females, *P*=0.0429; one way ANOVA with Dunnett's multiple comparison test). Body length was decreased by ∼5% in *Zfp469*^BCS/BCS^ mice relative to wild-type age- and sex-matched littermates (Fig. 2B), but this difference was not statistically significant at 3 or 6 months of age. There was no difference in eye size, measured using manual calipers, between genotypes in females at 3 months of age [average eye length from the front of the cornea to the posterior: ^+/+^_,_ 3.34±0.13 mm (mean±s.d., *n*=3), ^BCS/BCS^, 3.33±0.13 mm (mean±s.d., *n*=2)]. Other than the reduction in bodyweight, heterozygous and homozygous *Zfp469*^BCS^ mice were viable and fertile, with each genotype arising in the expected frequencies from heterozygote x heterozygote crosses (Table S2).

Expression of *Zfp469* in corneal keratocytes freshly isolated from corneas pooled by genotype was assessed by RT-qPCR using primers specific for *Zfp469* or for the V5-tag insertion. Relative *Zfp469* expression was increased to 1.69±0.64 (mean±s.d.) in homozygotes [1.00±0.15 (mean±s.d.) in wild types] but was not significantly different between genotypes (Fig. 2D). All of the *Zfp469* transcript expressed in homozygous mutant and 55%±3.7% (mean±s.d.) of transcript in heterozygous keratocytes contained the V5 sequence, showing that the mutant transcript is expressed at a similar level to the wild-type transcript. This is consistent with a premature stop codon in the single coding exon of Zfp469, resulting in the production of a truncated protein rather than the destruction of the transcript by nonsense-mediated decay (NMD; Fig. 2D).

### *Zfp469*^BCS^ mice recapitulate ophthalmic characteristics of BCS

**Fig. 3.**
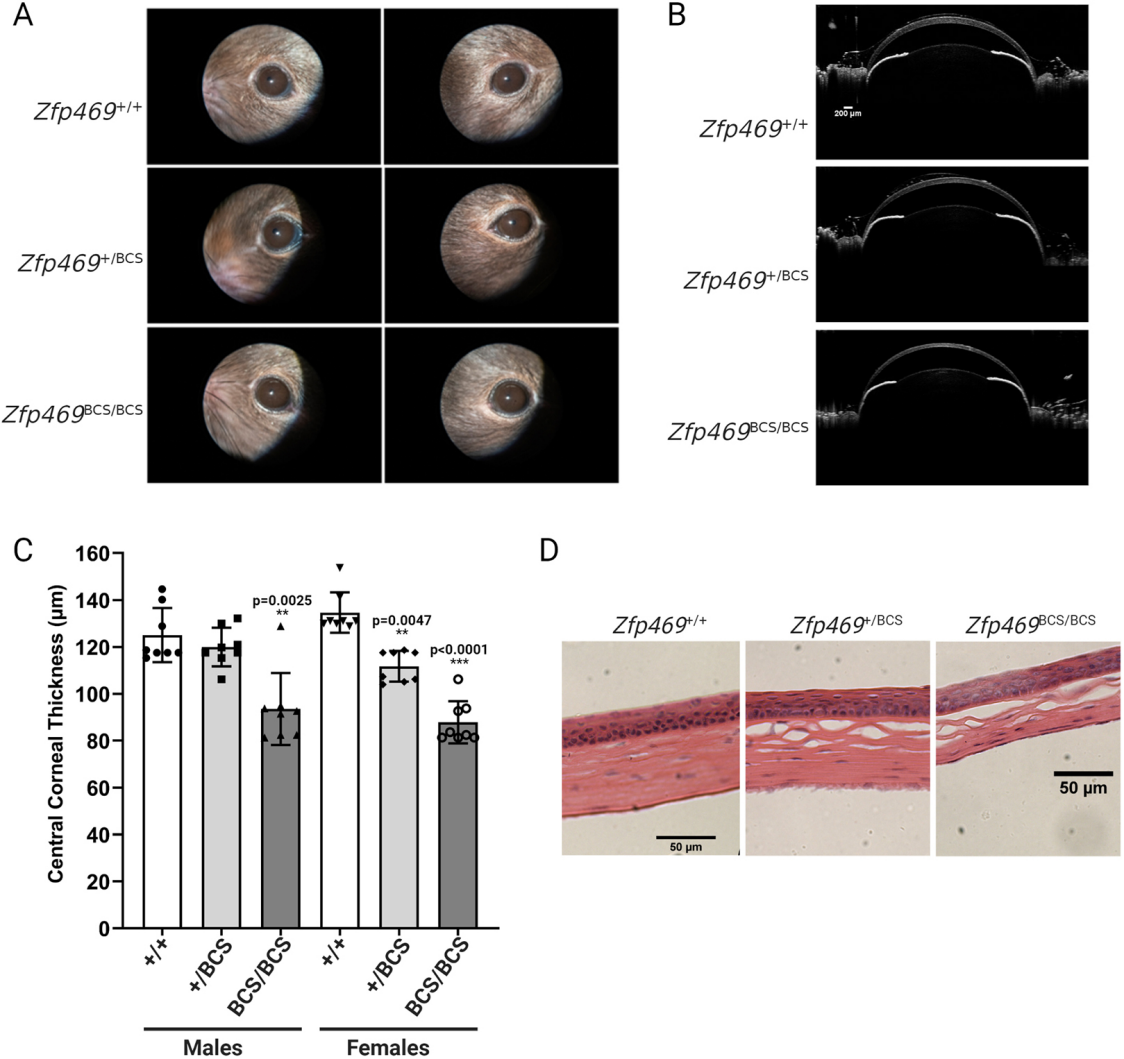
**Corneal thinning in *Zfp469*^BCS/BCS^ mice as a result of decreased stromal thickness.** (A) Corneal opacity was not observed in 3-month-old *Zfp469*^BCS/BCS^ mice examined by slit lamp. (B) AS-OCT of 3-month-old *Zfp469*^BCS/BCS^ mice demonstrated extreme thinning of the central and peripheral cornea compared to *Zfp469*^+/+^ and *Zfp469*^+/BCS^ age- and sex-matched mice. Scale bars: 200 µm. (C) CCT was decreased in *Zfp469*^BCS/BCS^ mice (*n*=4 for each genotype and sex; *P*=0.0025 in males, *P*<0.0001 in females, *P*<0.0001 for sexes combined; one-way ANOVA with Tukey's multiple comparison test). **P<0.01, ***P<0.001. Data are mean±s.d., with individual measurements from each eye shown by data points. (D) H&E staining of corneal sections from 3-month-old mice revealed visibly thinner corneal stroma in *Zfp469*^BCS/BCS^ mice.

Clinical characteristics of BCS include thinning of the cornea and sclera, myopia and refractive errors, keratoconus or keratoglobus, corneal rupture and vision loss. We performed ophthalmic phenotyping, including slit lamp examination, anterior-segment optical coherence tomography (AS-OCT), histology and immunostaining to determine whether the premature stop codon introduced into *Zfp469* led to features of BCS in the mouse. At 3 months of age, by which stage the cornea is fully developed (Hanlon et al., 2011), slit lamp examination revealed no gross corneal opacity or abnormality in homozygous mice (Fig. 3A). However, AS-OCT (Fig. 3B) showed that homozygous mice have extremely thin corneas relative to age- and sex-matched wild-type animals, with a mean reduction of 39.1±4.4 µm (mean±s.e.m.) in CCT (*P*=0.0025 in males, *P*<0.0001 in females, and *P*<0.0001 for sexes combined; one way ANOVA with Tukey's multiple comparison test; Fig. 3C). This corresponds to a 30% reduction in CCT. Thickness of the peripheral cornea was also significantly reduced by ∼25% [in female homozygous mice, average 144.6±15.3 µm (mean±s.d.), *n*=4; in wild-type females, 187.9±16.2 µm (mean±s.d.), *n*=3, *P*=0.0335], consistent with generalised thinning of the cornea as is seen in BCS.

Hematoxylin and Eosin (H&E) staining to examine corneal morphology confirmed that the multilayered epithelium appeared normal, as did the intact endothelium (Fig. 3D). However, the corneal stroma, which normally accounts for 90% of the thickness of a healthy cornea in humans and ∼60% of thickness in mice (Hanlon et al., 2011), was markedly thinner in homozygotes. Extensive shearing was observed between lamellae relative to that seen in wild-type cornea processed for staining in the same way (Fig. 3D).

### Corneal thinning in *Zfp469*^BCS^ mice is apparent during corneal development and is not progressive

**Fig. 4.**
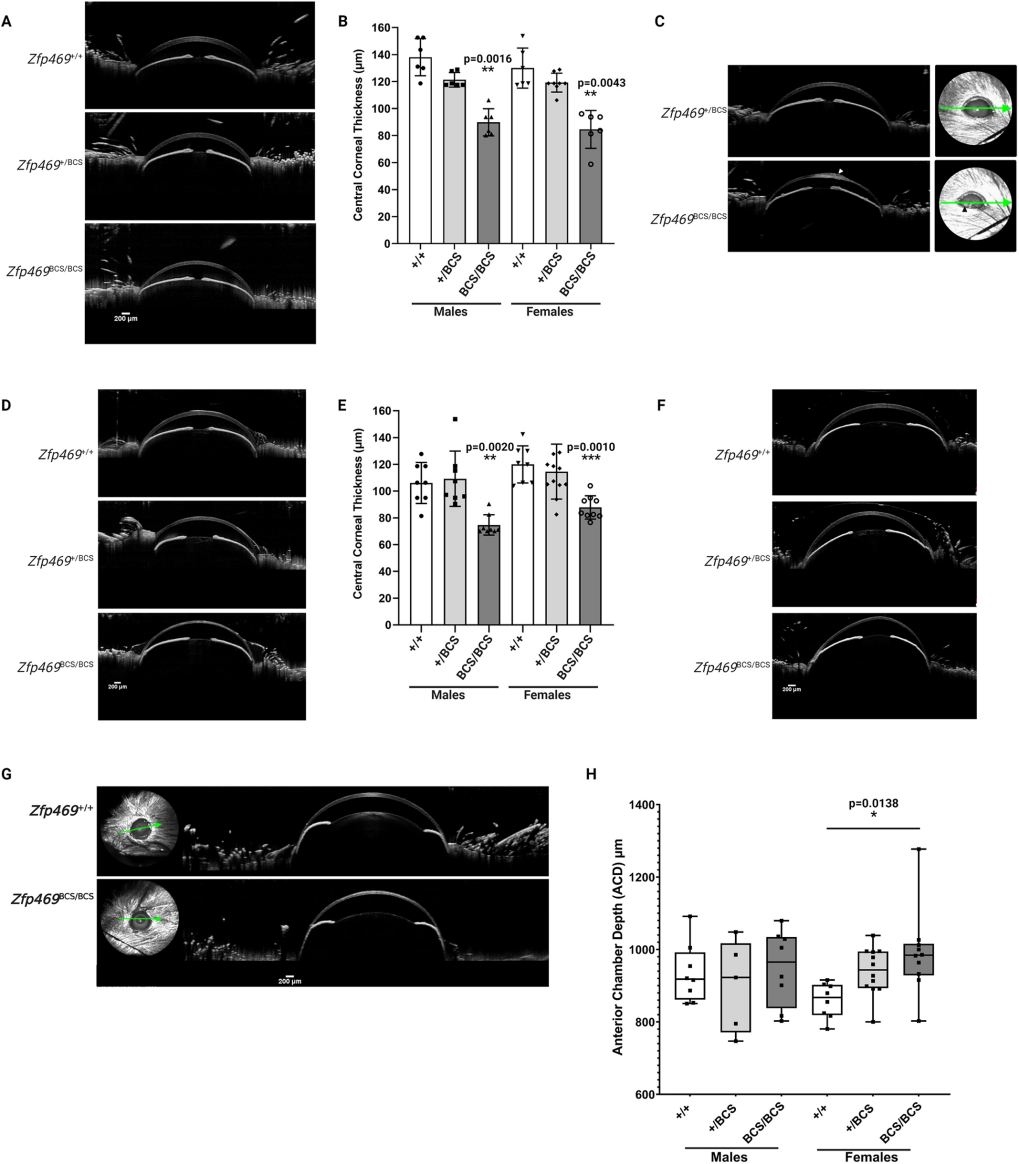
**Corneal thinning in *Zfp469*^BCS^ mice is apparent during corneal development and is not progressive.** (A) Extreme thinning of the cornea in 1-month-old *Zfp469*^BCS/BCS^ mice compared to *Zfp469*^+/+^ and *Zfp469*^+/BCS^ age- and sex-matched mice was observed by AS-OCT. (B) At 1 month of age, CCT was significantly decreased in *Zfp469*^BCS/BCS^ mice (*P*=0.0016 in males, *P*=0.0043 in females, *P*<0.0001 for sexes combined, one-way ANOVA with Tukey's multiple comparison test). *N*=3 for each genotype in males; *n*=3 for *Zfp469*^+/+^ and *Zfp469*^BCS/BCS^, *n*=4 for *Zfp469*^+/BCS^ in females. Data are mean±s.d., with individual measurements from each eye shown by data points. (C) Unilateral corneal opacity observed in one eye from a *Zfp469*^BCS/BCS^ female at 1 month of age resembled corneal edema (white arrowhead). Green arrows indicate the direction and position of the OCT scans. (D) CCT at 6 months of age remained similar to CCT measured at 1 and 3 months of age, with significant central and peripheral thinning apparent only in *Zfp469*^BCS/BCS^ mice. (E) CCT was significantly decreased in *Zfp469*^BCS/BCS^ mice at 6 months of age (*P*=0.0020 in males, *P*=0.0010 in females, *P*<0.0001 for sexes combined, one-way ANOVA with Tukey's multiple comparison test). *N*=4 for each genotype in males; *n*=4 for *Zfp469*^+/+^, *n*=6 for *Zfp469*^+/BCS^, *n*=5 for *Zfp469*^BCS/BCS^ in females. Data are mean±s.d., with individual measurements from each eye shown by data points. (F) Corneal distortion was seen by AS-OCT after the application of eye drops in *Zfp469*^BCS/BCS^ eyes, but not in heterozygous or wild-type sex-matched littermates, suggesting a loss of biomechanical strength in the mutant corneas. (G) Globular protrusion of the eye, resembling keratoglobus, was observed after dilation of the pupil in *Zfp469*^BCS/BCS^ female mice, but not in male mice or heterozygous or wild-type sex-matched controls. (H) ACD in the eyes of *Zfp469*^BCS/BCS^ female mice, measured from AS-OCT images, was significantly increased compared to heterozygous or wild-type sex-matched controls after dilation of the pupil. *P*=0.0047, Welch's ANOVA test). *n*=4 for *Zfp469*^+/+^, *n*=3 for *Zfp469*^+/BCS^, *n*=4 for *Zfp469*^BCS/BCS^ in males; *n*=4 for *Zfp469*^+/+^, *n*=6 for *Zfp469*^+/BCS^, *n*=5 for *Zfp469*^BCS/BCS^ in females. Box plot shows all data, with individual measurements from each eye shown by data points. Whiskers show minimum to maximum values. *P<0.05, **P<0.01, ***P<0.001.

In some clinical reports, the corneal thinning observed in BCS patients is described as progressive and corresponds with an increasing risk of spontaneous rupture of the cornea or sclera. We sought to determine whether the corneal thinning observed in the *Zfp469*^BCS/BCS^ mice at 3 months was established earlier, during development of the cornea, and whether thinning of the stroma was progressive. At 1 month of age, AS-OCT (Fig. 4A) revealed that the central corneas of homozygous animals were, on average, already 46.8±5.6 µm (mean±s.e.m.) thinner than wild-type sex- and age-matched controls (Fig. 4B; *P*=0.0016 males, *P*=0.0043 females, *P*<0.0001 for sexes combined; one way ANOVA with Tukey's multiple comparison test) – a 35% reduction in CCT. Fig. 4C shows a unilateral corneal opacity observed in one eye from a *Zfp469*^BCS/BCS^ female at 1 month of age (*n*=12 studied). Optical coherence tomography (OCT) revealed swelling of the corneal stroma consistent with an edema. It was not possible to determine whether this was a result of injury leading to rupture of Descemet's membrane but there was no evidence of injury or infection. No such accumulation of fluid within the cornea was observed in 26 heterozygous or wild-type eyes at 1 month of age, or in any older mice.

Subsequent investigation of corneal thickness at 6 months of age also showed a 30% reduction in CCT, with mean CCT 31.4±5.2 µm (s.e.m.) thinner in *Zfp469*^BCS/BCS^ (Fig. 4D,E; *P*=0.0023 males, *P*=0.001 females, *P*<0.0001 combined). Peripheral corneal thickness (PCT) was also significantly reduced [in female homozygous mice, average 125.99±14.1 µm (mean±s.d.), *n*=4; in wild-type females, 166.9±21.8 µm (mean±s.d.), *n*=3; *P*=0.0286]. The degree of thinning observed at 1, 3 and 6 months consistently reduced CCT by ∼30% relative to wild-type age- and sex- matched mice. The corneas of individual mice with homozygous BCS mutation in Zfp469, followed from 3 months of age, did not progressively thin up to 6 months of age.

The thin corneas of homozygous mutant mice up to 9 months of age were not prone to perforation; no animals were affected by corneal damage under normal housing conditions. However, the *Zfp469*^BCS/BCS^ corneas were more easily distorted by the application of a viscous liquid drop, intended to prevent the eye from drying out during OCT, than wild-type controls at both 3 and 6 months of age (Fig. 4F). We also measured anterior chamber depth (ACD) in AS-OCT images obtained from female mice at 6 months of age. Without dilation of the pupils, ACD in *Zfp469*^+/+^ was 977.1 µm±32.9 (mean±s.d., *n*=3), in *Zfp469*^+/BCS^ 1002.9 µm±55.1 (mean±s.d., *n*=5), and in *Zfp469*^BCS/BCS^ 1016.5 µm±32.1 (mean±s.d., *n*=4). This difference was not significant but suggested there may be a trend towards increased ACD in the eyes of homozygous mutant mice. Subsequently, when the pupils of the same animals were dilated by the administration of tropicamide and phenylephrine hydrochloride prior to performing AS-OCT, female *Zfp469*^BCS/BCS^ showed a bulging eye phenotype consistent with keratoglobus (Fig. 4G), a condition in which generalised corneal thinning results in globular protrusion of the eye. This was not observed in male mice, or in wild-type females. ACD was determined from OCT images after pupil dilation, confirming a significant increase in ACD in *Zfp469*^BCS/BCS^ females relative to wild-type controls at 6 months of age (Fig. 4H; Welch's ANOVA test, *P*=0.0138). Interestingly, the ACD of homozygous mutant eyes decreased by only 2.6% after dilation of the pupil. This contrasted with wild-type ACD, which was reduced by 12% on average relative to ACD determined without dilation of the pupil. Together with the specific thinning of the stroma revealed by H&E staining, these data are indicative of a loss of biomechanical strength of the corneal stroma, resulting in impaired resistance to both internal and external forces.

### Collagen type I, but not collagen type V, is less abundant in the *Zfp469*^BCS/BCS^ cornea, and fibril architecture is altered

**Fig. 5.**
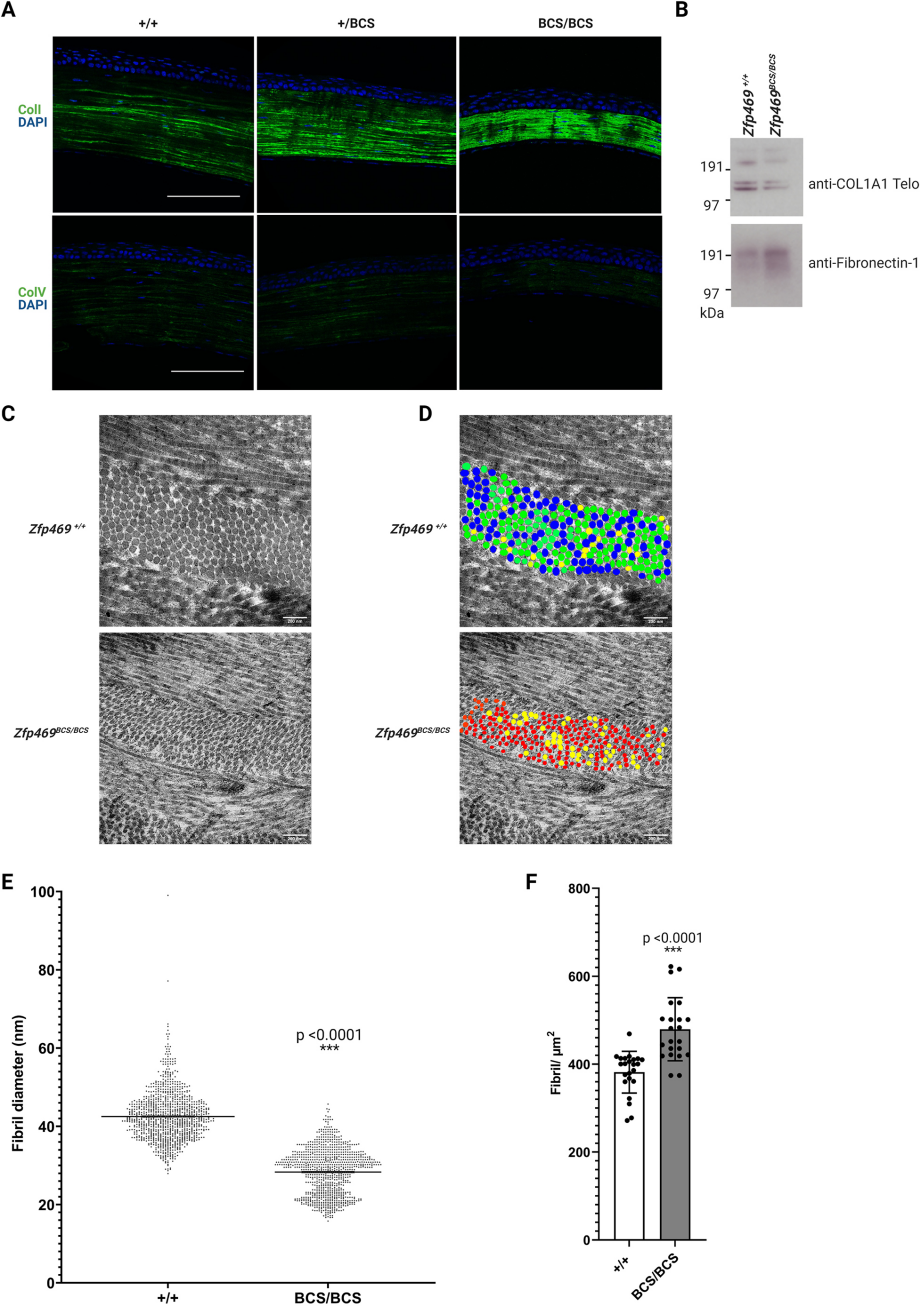
**Collagen type I is less abundant and affects fibril architecture in the stroma of *Zfp469*^BCS/BCS^ corneas.** (A) Collagen type I and type V localise to the corneal stroma in 6-month-old mice, with decreased stromal area apparent in *Zfp469*^BCS/BCS^ compared to wild-type and heterozygous age- and sex-matched samples subjected to immunofluorescence microscopy. Nuclei are stained blue with DAPI, and collagen type I and type V are stained green. (B) Western blot showing that Col1a1 is decreased in *Zfp469*^BCS/BCS^ corneas at 2 months of age. Fibronectin-1 was used as a loading control. (C) Representative TEM of the cornea anterior stroma showed regular fibril organisation and packing of lamellae in mice at 9 months of age. (D) Colour-coded images of collagen fibrils in the images shown in C, showing that fibrils in wild-type corneal stroma are mostly green (40-49 nm), whereas homozygous mutant fibrils in the anterior stroma are mostly <29 nm (red). Fibrils with a diameter of 30-39 nm are coded yellow and those with a diameter of 50-59 nm are coded blue. (E) Fibril diameter was significantly decreased in the anterior stroma of *Zfp469*^BCS/BCS^ mice (*P*<0.0001, unpaired two-tailed Student's *t*-test). The range of diameters in 1059 measurements made in sections from three wild-type mice was 27.93-99.05 nm, and in 912 measurements made in sections from *Zfp469*^BCS/BCS^ mice the range was 15.79-45.70 nm. (F) The smaller fibrils in *Zfp469*^BCS/BCS^ stroma were more tightly packed, as shown by significantly increased fibril density in the corneal stroma of *Zfp469*^BCS/BCS^ mice, measured in 23 sections from three wild-type mice, and 22 sections from *Zfp469*^BCS/BCS^ mice (*P*<0.0001, unpaired two-tailed Student's *t*-test). ***P<0.001. Error bars are mean±s.d. Scale bars: 100 µm (A); 200 nm (C,D).

The corneal stroma is a collagenous ECM generated by resident keratocytes. Having observed thinning of the corneal stroma in *Zfp469*^BCS**/**BCS^, we next analysed the amount of collagen type I and collagen type V in the corneal stroma. In wild-type corneas at 6 months of age, immunostaining for both collagen type I and type V showed uniform distribution throughout the stroma, each having a characteristic appearance (Fig. 5A). Collagen type I (ColI) staining appeared to demarcate linear structures within the stroma; collagen type V staining (ColV) staining had a more punctate appearance within the linear structures. This is consistent with their roles in the assembly of collagen fibrils in the cornea, with collagen type I the predominant fibril-forming collagen, and collagen type V having an important role in the nucleation of collagen type I fibrils. In *Zfp469*^BCS/BCS^ cornea sections, the staining of collagen type I and type V was strikingly confined to a much thinner stroma. Both collagen type I and type V staining in homozygous mutant corneas appeared to be more densely packed than in heterozygous or wild-type corneas, but quantification of fluorescent signals determined that total intensity was not significantly different to wild-type controls (Fig. 5A).

Less Col1a1 Telo collagen was detected by immunoblotting in the lysate of six pooled *Zfp469*^BCS/BCS^ corneas compared to wild type, relative to the amount of fibronectin-1 (Fig. 5B). These data suggested that collagen production or fibrillogenesis may be affected in BCS corneas, leading us to assess the organisation of the corneal stroma and fibril structure in mice at 9 months of age using transmission electron microscopy (TEM) (Fig. 5C). Images obtained from the anterior central cornea showed the lamellae were cylindrical and regularly arranged in both wild-type and mutant corneas, but revealed a striking difference in fibril diameter between three wild-type and three *Zfp469*^BCS/BCS^ samples. Morphometric analysis revealed that the diameters of cross-sectional collagen fibrils were significantly smaller in *Zfp469*^BCS/BCS^ stroma than in wild type [mean fibril diameter, 28.32 nm±5.93 nm (s.d.), compared with 42.53 nm±6.32 nm (s.d.); *P*<0.0001]. In anterior stroma of individual wild-type and *Zfp469*^BCS/BCS^ corneas, there was a narrow distribution of fibril size, as shown in the colour-coded images in Fig. 5D. The distribution of collagen fibril diameter in three corneas per genotype group is shown in Fig. 5E. Collagen fibril diameter in the anterior stroma was, on average, 33% reduced in *Zfp469*^BCS/BCS^ mice. In addition, an increased number of collagen fibrils were present in a given area of mutant stroma compared to wild type, with fibril density (fibrils/µm^2^) of the smaller diameter fibrils being significantly increased from 381.7±47.46 (mean±s.d.) fibrils/µm^2^ to 479.3±71.77 (mean±s.d.) fibrils/µm^2^. This corresponds to an increase of 25.6% (Fig. 5F, mutant versus wild type, *P*<0.0001) in the stroma of homozygous mutant corneas.

Extraocular features of BCS may include generalised connective tissue dysfunction, leading to joint hypermobility, easy bruising and soft doughy skin (Burkitt Wright et al., 2013). During euthanasia, there were three instances of tail degloving (separation of the skin and subcutaneous tissues) in *Zfp469*^BCS/BCS^ adult mice. No such degloving events occurred for wild-type or heterozygous mice. This suggests that LOF of Zfp469 may compromise the biomechanical strength of the skin or underlying connective tissue. Given that collagen type I is the major component of human skin (85-90%), with collagen type III and type V accounting for 8-11% and 2-5% of total collagen, respectively, we sought to determine whether the skin was affected in *Zfp469*^BCS/BCS^ mice. H&E staining of sections of tail skin from 6-month-old mice revealed decreased thickness of the dermal and subcutaneous adipose layers in homozygous mutants (Fig. S1A). Masson's trichrome staining of collagen in the dermis revealed that the skin of *Zfp469*^BCS/BCS^ mice contains less collagen than wild-type animals (Fig. S1). This appears to arise from a decreased abundance of collagen type I in the dermis, as shown in a representative image of adult tail skin in Fig. S1C. In keeping with our earlier observation from corneal sections, collagen type V staining did not alter with genotype (Fig. S1D), suggesting that deficiency of collagen type I underlies both the ocular and extraocular features of BCS.

### A primary cell model of stromal composition in BCS

**Fig. 6.**
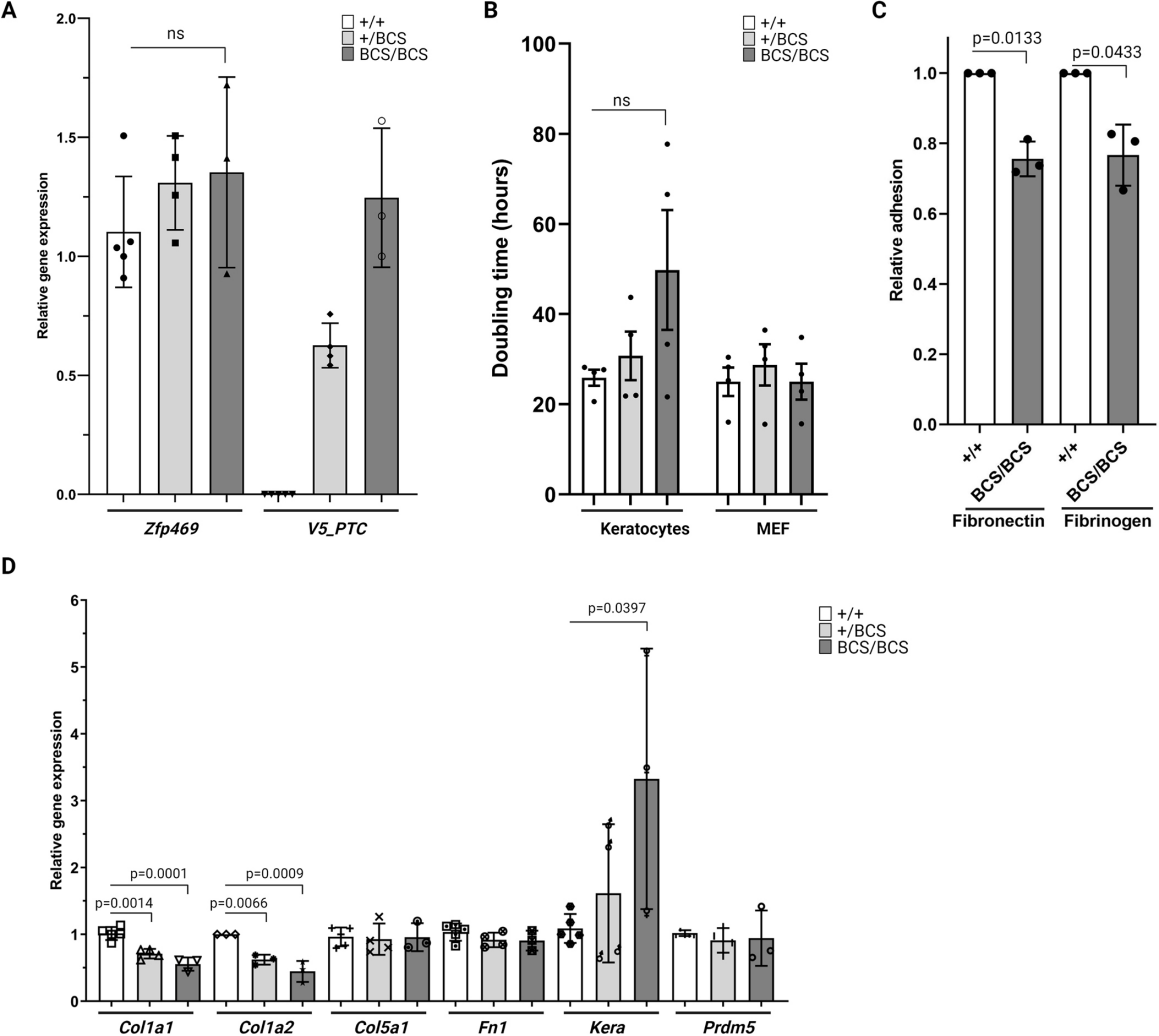
**Mutation in Zfp469 affects the expression of ECM genes by corneal stroma fibroblasts in culture.** (A) Relative expression of *Zfp469* in primary keratocytes cultures, determined by qRT-PCR, was not significantly different between genotypes. There was an allele dosage effect on the expression of *Zfp469* transcript containing the V5 tag and premature termination codon (V5_PTC), with *Zfp469*^+/BCS^ expressing half as much of this transcript as *Zfp469*^BCS/BCS^ keratocytes lines (*n*=5 for *Zfp469*^+/+^, *n*=4 for *Zfp469*^+/BCS^, *n*=3 for *Zfp469*^BCS/BCS^; one-way ANOVA with Dunnett's multiple comparison test). Data are mean±s.d., with the average of assays performed in triplicate from each sample shown by data points. (B) Primary keratocytes and MEFs were seeded at known density at passage three, and the doubling time was determined by cell counting after 96 h in culture. No significant difference in doubling time was observed, although *Zfp469*^BCS/BCS^ keratocytes showed the greatest variability and longest average doubling time. Data are mean±s.e.m., with the doubling time of individual cell lines shown by data points. (C) Relative adhesion of *Zfp469*^BCS/BCS^ cells to fibronectin and fibrinogen-coated wells after 45 min compared to litter-mate wild-type keratocytes was significantly decreased [wild-type adhesion normalised to 1, average adhesion of *Zfp469*^BCS/BCS^ cells to fibronectin was 0.755±0.049 (mean±s.d.) (*P*=0.0133, Welch's *t*-test), and average adhesion of *Zfp469*^BCS/BCS^ cells to fibrinogen was 0.766±0.087 (mean±s.d.), *P*=0.0433, Welch's *t*-test]. Data are presented as relative adhesion (wild-type absorbance 560 nm/homozygous absorbance 560 nm)±s.d. Three cell lines for each genotype were used. (D) Relative expression of key genes in the ECM and in BCS was assessed by qRT-PCR in primary keratocytes. *Col1a1*, *Col1a2* and *Kera* showed a significant *Zfp469*^BCS^ allele dosage effect on gene expression. *Hprt* and *Gapdh* were used to normalise the amount of mRNA, and data were analysed using the 2^−ΔΔCT^ method for relative quantitation. Fold change is relative to *Zfp469*^+/+^. Data are mean±s.d., with the average of assays performed in triplicate from each sample shown by data points (*n*=5 for *Zfp469*^+/+^, *n*=4 for *Zfp469*^+/BCS^, *n*=3 for *Zfp469*^BCS/BCS^; one-way ANOVA with Dunnett's multiple comparison test). ns, not significant.

Keratocytes synthesise components of the healthy stroma and repair tissue damage. There was no significant difference in the number or density of stromal keratocytes in *Zfp469*^BCS/BCS^ corneal sections visualised by DAPI staining of keratocyte nuclei compared to wild-type controls at 6 months of age (Fig. 5A). To determine how loss of function of Zfp469 affects the population of stromal keratocytes, we established primary mouse keratocyte cultures from corneas taken from neonatal pups at postnatal day (P)2. At this stage of development of the cornea, keratocytes are still dividing and have yet to become quiescent as in mature corneas. From these cultures of corneal stroma fibroblasts, we investigated the gene expression profile in *Zfp469*^BCS/BCS^ primary keratocytes. First, using RT-qPCR, we established that the expression of *Zfp469* closely reflected that seen in freshly isolated keratocytes, with no significant difference in *Zfp469* gene expression between genotypes (Fig. 6A). Half of the transcript detected in heterozygous cell lines carried the V5 tag and premature stop codon CRISPR/Cas9-mediated gene edit; in homozygotes, 100% of the *Zfp469* transcript carried this mutation.

The proliferative ability of the BCS keratocytes *in vitro*, determined using the population doubling time for each cell line seeded at the same density at passage three, was not significantly altered by the LOF mutation in Zfp469 (Fig. 6B). However, three out of four *Zfp469*^BCS/BCS^ primary keratocyte lines did proliferate more slowly than wild-type lines isolated from littermates, suggesting that there may be an impact on the rate of proliferation that we are underpowered to detect. The mean doubling time for *Zfp469*^+/+^ primary keratocytes was 25.86 h±1.77 (s.e.m.) compared to 49.80 h±13.31 (s.e.m.) for *Zfp469*^BCS/BCS^ keratocytes (*n*=4 for each genotype; Fig. 6B). Of further note, the two mutant cell lines that proliferated most slowly failed to proliferate well beyond passage four. This was not observed for wild-type cell lines generated from littermates or for MEFs, which showed no variability in doubling time by genotype (Fig. 6B). This suggests that there might be a genotype- and cell-type-specific effect of *Zfp469* mutation on cell proliferation.

The adhesion of keratocytes to ECM is key to establishing and maintaining the structural integrity of the cornea (Parapuram et al., 2011). We sought to determine the impact of LOF of Zpf469 on the adhesion of primary keratocytes to a variety of ECM components, including collagen type I, collagen type IV, fibrinogen, fibronectin and Laminin. This revealed that *Zfp469*^BCS/BCS^ keratocytes adhere less efficiently to fibrinogen (*P*=0.0433) and fibronectin (*P*=0.0133) compared to wild-type controls (Fig. 6C). Defective adhesion to fibronectin, an ECM protein with known roles in the maintenance of tissue architecture and wound healing, may impact upon the arrangement and organisation of collagen fibrils in the stroma.

The expression of *Prdm5*, in which mutations cause BCS type 2, and key components of the stromal ECM was investigated using qPCR (Fig. 6D). *Prdm5* expression was unchanged in *Zfp469*^BCS^ primary keratocytes. However, a significant impact of *Zfp469*^BCS^ allele dosage on the expression of *Col1a1* and *Col1a2*, the genes encoding the alpha 1 and alpha 2 chains of collagen type I that associate to form the triple helix of individual collagen type I, was uncovered. The relative expression of both *Col1a1* and *Col1a2* was reduced by ∼50% in homozygous mutant cell lines compared to wild-type lines (*Col1a1*, adjusted *P*=0.0001; *Col1a2*, adjusted *P*=0.0009; one-way ANOVA with Dunnett's multiple comparison test), and by ∼25% in heterozygous primary keratocytes (*Col1a1*, adjusted *P*=0.0014; *Col1a2*, adjusted *P*=0.0066; Fig. 6D). The expression of *Col5a1* in BCS keratocytes in culture was not significantly altered compared to wild-type cell lines, in agreement with our observations from corneal section staining. Expression of *Kera*, a key keratocyte marker, also showed an allele dosage effect of mutation of Zfp469, increasing 1.5-fold in ^+/BCS^ and 3-fold in ^BCS/BCS^ keratocyte cell lines (adjusted *P*=0.0397; one-way ANOVA with Dunnett's multiple comparison test). *Kera* encodes keratocan, a cornea-specific keratan sulfate proteoglycan (KSPG) belonging to the small leucine-rich proteoglycan (SLRP) gene family, and plays an important role in the regulation of collagen fibril spacing and arrangement in the mature cornea. These data point to a dysfunctional gene expression profile in mutant keratocyte cells that may affect the composition and assembly of the stromal ECM.

**Fig. 7.**
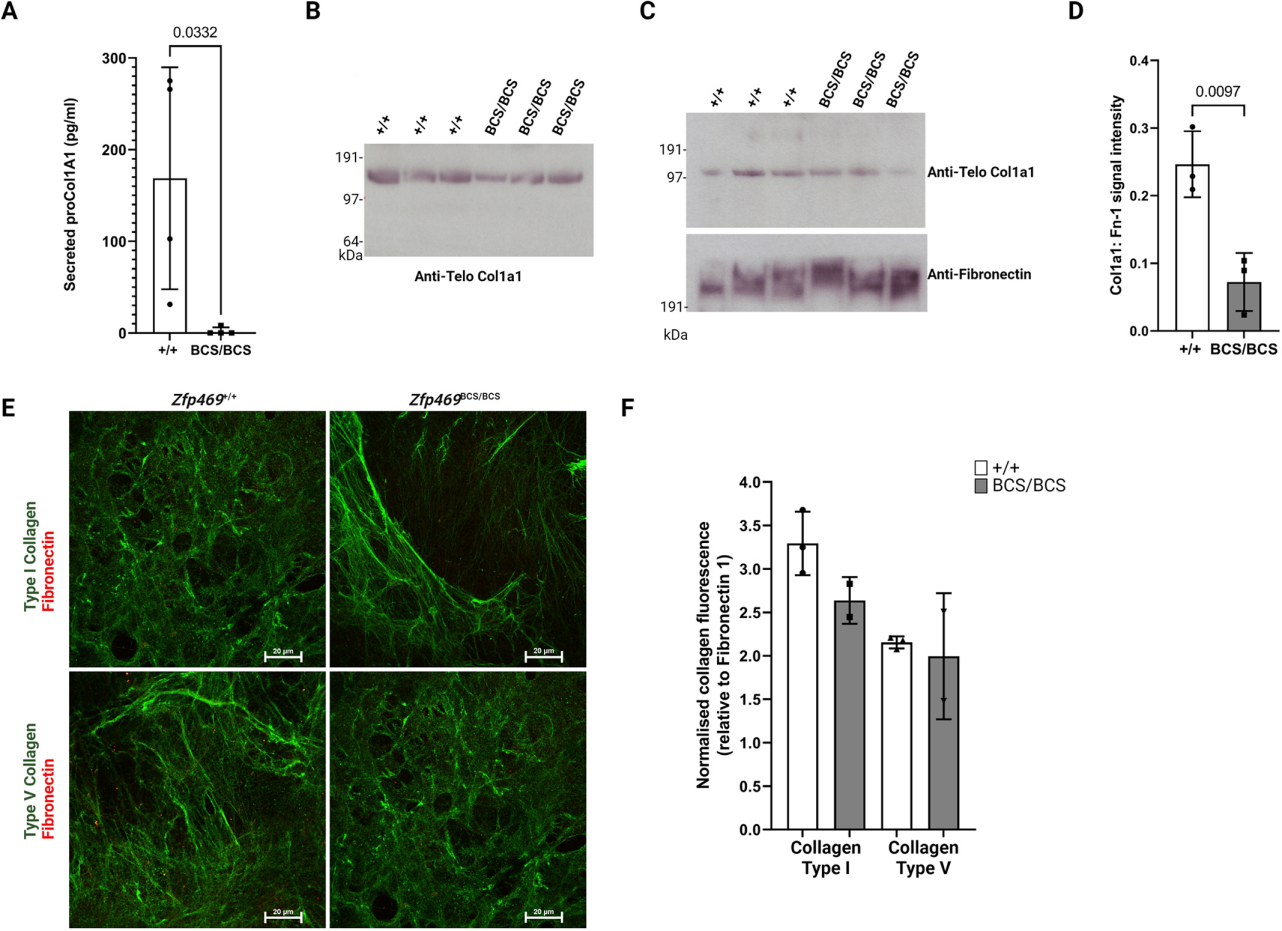
**Secretion and deposition of collagen type I by primary keratocytes is impaired when Zfp469 is mutated.** (A) The concentration of proCol1a1 in serum-free medium after 7 days in culture, as measured by ELISA, showed that secretion of proCol1a1 into culture medium by confluent *Zfp469*^BCS/BCS^ primary keratocytes was significantly impaired compared to wild-type cells. Data are mean±s.d. with the proCol1a1 concentration of individual cell lines shown by data points (*n*=4 for *Zfp469*^+/+^, *n*=4 for *Zfp469*^BCS/BCS^; unpaired two-tailed Student's *t*-test). (B) Representative western blot of the serum-free conditioned medium samples used in ELISA that were probed with an anti-Telo Col1a1 antibody that detects prepro, pro and mature Col1a1 shows decreased Col1a1 in the medium of *Zfp469*^BCS/BCS^. (C) CDMs generated by primary keratocytes were decellularised and then denatured and reduced prior to western blotting. The CDM of *Zfp469*^BCS/BCS^ contained less Telo Col1a1 than wild-type CDM. Fibronectin was used as a loading control. (D) Quantification of Col1a1 signal normalised to fibronectin for CDMs generated from three *Zfp469*^+/+^ and three *Zfp469*^BCS/BCS^ by densitometry shows a significant decrease in the abundance of Telo Col1a1 in mutant cell lines (*n*=3 per group, *P*=0.0097; unpaired two-tailed Student's *t*-test). (E) Representative immunofluorescence images of CDMs generated by primary keratocytes after 20 days exposure to ascorbic acid-containing medium, and stained for collagen type I (green), collagen type V (green) and fibronectin (red). Scale bars: 20 µm. (F) Immunofluorescence staining intensity for collagen type I and collagen type V was normalised to fibronectin signal for CDMs from three *Zfp469*^+/+^ and two *Zfp469*^BCS/BCS^ cell lines, using five non-overlapping images from each sample. Data are mean±s.d. with the average normalised collagen staining intensity of individual cell lines shown by data points. Deposition of collagen type V did not differ between genotypes but collagen type I deposition was decreased in *Zfp469*^BCS/BCS^ CDMs.

We next investigated the secretion and deposition of collagen type I by primary keratocytes. The amount of proCol1a1 secreted into the medium by confluent *Zfp469*^BCS/BCS^ primary keratocytes maintained in serum-free medium for 7 days was significantly reduced compared to wild-type cells (Fig. 7A; *P*=0.0332; *t*-test). Immunoblotting of the same serum-free conditioned medium samples using an anti-Telo Col1a1 antibody confirmed that *Zfp469*^BCS/BCS^ primary keratocytes secrete less Col1a1 (Fig. 7B), with densitometry revealing a 43±9% (mean±s.d.) depletion of Col1a1 secreted relative to wild-type samples. Cell-derived matrices (CDMs) from homozygous mutant and wild-type primary keratocytes were generated to study ECM deposition *in vitro*. Keratocytes were seeded onto gelatin-coated plates or coverslips and, once confluent, were treated with ascorbic acid for 20 days to induce deposition of a collagen-rich matrix. Immunoblot analysis of the CDMs collected following decellularisation under reducing and denaturing conditions revealed that less collagen type I was deposited by mutant cell lines (Fig. 7C). The expression of the fibronectin-1 gene (*Fn1*) was unaffected by Zfp469 mutation (Fig. 6D). Using densitometric analysis of Col1a1 Telo signal normalised to fibronectin-1 (Fig. 7D), *Zfp469*^BCS/BCS^ primary keratocytes deposited, on average, only 30% of Col1a1 to the CDM relative to wild-type cells (*P*=0.0097, *t*-test). Intact, decellularised CDMs were also stained with antibodies against collagen type I, collagen type V and fibronectin (Fig. 7E). Quantification of fluorescent signals in CDM generated by two *Zfp469*^BCS/BCS^ cell lines confirmed that collagen type I was deposited by *Zfp469*^BCS/BCS^ primary keratocytes, whereas collagen type V remained unchanged relative to wild type (Fig. 7F).

## DISCUSSION

BCS was first reported in 1968 (Stein et al., 1968) but it was another 40 years until mutations in *ZNF469* were identified as a cause of this rare condition. Since then, 30 pathogenic mutations in *ZNF469* have been identified, the majority of these resulting in premature stop codons preceding zinc-finger domains. The precise mechanism by which biallelic LOF mutations result in generalised connective tissue dysfunction, and why the most severe manifestation occurs in the cornea and sclera, has remained unclear. This study is the first description of a mouse model of BCS, in which we created a premature stop codon in the orthologous mouse gene *Zfp469* to recapitulate the human mutation p.Gly677*. Our results showed that this premature stop codon in the large single coding exon of *Zfp469* does not lead to NMD of the mutant transcript and will likely lead to the generation of a truncated protein. Despite incorporation of an in-frame V5 tag, we were unable to detect a V5-tagged protein by western blotting or immunostaining of tissue samples or fixed primary keratocytes. This suggests that the protein may be expressed at very low levels, the mutated V5 is not recognised or that the V5 tag is inaccessible. Although the mutant protein could not be detected, disrupting Zfp469 clearly perturbs the development of the corneal stroma in mouse, resulting in thinning of the corneas by one-third in homozygous mutant eyes at 1 month of age. At 3 and 6 months of age, the mature cornea of *Zfp469*^BCS/BCS^ eyes remain 30-35% thinner than wild types, suggesting that, in mice, stromal thinning is not progressive.

In humans with BCS, a progressive loss of stromal depth has been described previously (Burkitt Wright et al., 2013). BCS patients suffer from progressive keratoconus or keratoglobus, with alteration of corneal shape due to distortion observed over 1 year (Ramappa et al., 2014) or longer (Skalicka et al., 2020). Corneal edema resulting from breaks in the Descemet's membrane may occur, and extremely thin corneas are prone to rupture after minor injury (Burkitt Wright et al., 2013). No longitudinal studies of CCT in BCS have been reported, probably due to the young age at which corneal perforations occur {mean age of published cases with rupture, 4.3 years of age [range 1.5-19 years (Walkden et al., 2019)]}. None of our BCS mice, aged up to 9 months, suffered from cornea rupture, and only one case of corneal edema was recorded. This apparent difference in corneal fragility may reflect that the stroma of the mouse cornea contributes only 60% of total thickness compared to 90% in humans, and underlies a proportionally thicker corneal epithelial layer (Hanlon et al., 2011). The epithelial layer appears unaffected in our model. Despite these anatomical differences, the bulging eye phenotype observed in our BCS mouse model following dilation of the pupil closely resembled keratoglobus. Furthermore, the corneas of homozygous mutant mice showed a decreased ability to withstand corneal strain, deforming after either application of an external eye drop or by dilation of the pupil. This is consistent with a loss of biomechanical strength in the corneal stroma when Zfp469 function is lost, and is in striking contrast to the corneas of wild-type mice, which were able to maintain normal corneal curvature under the same pressure load. This study is the first to show a direct impact of LOF of Zfp469 upon cornea biomechanical strength. However, the genetic association of regulatory variants for *ZNF469* with corneal resistance factor in genome-wide association studies indicates that ZNF469 also plays a role in determining the biomechanical properties of the cornea in humans (Jiang et al., 2020; Khawaja et al., 2019; Simcoe et al., 2020).

Numerous studies have shown that disrupting the composition or organisation of the stromal ECM will affect the shape (Quantock et al., 2003) or strength (Chakravarti et al., 1998) of the cornea. Consistent with this, the corneal thinning caused by loss of Zfp469 appears to result from a ∼50% reduction in the expression of genes encoding collagen type I, *Col1a1* and *Col1a2*, by keratocytes. This is an important result, as the relative abundance of collagen type I and type V in the cornea appears key to the proper organisation of the stroma. Collagen composition in the cornea is unique among tissues, with fibrillar collagen in the stroma made up of ∼80-90% collagen type I and 10-20% collagen type V. Collagen type I assembles into heterotrimers of two alpha 1(I) chains and one alpha 2(I) chain, growing further into heterotypic fibrils with collagen type V. Non-helical terminal extensions of collagen type V protrude from the fibril (Linsenmayer et al., 1993), acting to nucleate fibril growth and determine fibril diameter. Strikingly, the expression of collagen type V remained unchanged in our primary keratocyte cells, in CDMs, and with staining of corneal sections from *Zfp469*^+/+^ and *Zfp469*^BCS/BCS^ mice, providing possible insight into the pathomechanisms underlying BCS caused by mutation in Zfp469.

Analysis of fibril diameter, density and organisation in the corneal stroma of our BCS model by TEM further implicated reduced collagen type I expression in corneal stromal thinning. The diameter of collagen fibrils was reduced by 33% in *Zfp469*^BCS/BCS^ mice compared to wild type, and fibril density was increased by 25%. This closely resembled the ultrastructural phenotype of the osteogenesis imperfecta (OI) mouse model resulting from homozygous mutation in *Col1a2* (oim; Chipman et al., 1993)*.* The CCT of *Col1a2^oim/oim^* corneas is 15% thinner than wild-type controls, and fibril diameter determined by TEM is also reduced by 15%. These features arise from impaired collagen fibrillogenesis as a result of loss of Col1a2 during the assembly of collagen type I heterotrimers (Dimasi et al., 2010). Notably, collagen type I homotrimers containing three units of Col1a1 have been observed in adult skin and in OI (Nicholls et al., 1984; Pace et al., 2008; Pihlajaniemi et al., 1984; Uitto, 1979). The concurrent decrease in expression of both *Col1a1* and *Col1a2* may explain the more severe corneal thinning observed in both the BCS model and in BCS compared to OI in humans.

Interestingly, the arrangement of fibrils within the lamellae of mature *Zfp469*^BCS/BCS^ corneas was regular and the organisation of parallel lamellae appeared normal. Indeed, corneal transparency was maintained throughout our study. A similar observation has been made in a previous report of *Col5a1* haploinsufficiency, in which heterozygous loss of *Col5a1* results in thinning of the mature mouse corneal stroma by 26%, resulting in 14% decrease in total collagen content and a 25% reduction in the number of fibrils in the stroma. Fibril diameter was increased and fibril density was reduced in this model of EDS, but no corneal opacity was observed (Segev et al., 2006). This is in contrast to Col5a1^Δst/Δst^ mice, in which targeted deletion of *Col5a1* in the corneal stroma results in the total absence of Col5a1 from the stroma, leading to larger and less uniform collagen fibrils and extensive corneal hazing (Sun et al., 2011). These data confirm, firstly, that regular packing of a homogeneous population of fibrils with a narrow distribution of diameters is key to transparency (Hassell and Birk, 2010), and secondly, that the ratio between collagen type I and type V is crucial for the assembly of a well-ordered and mechanically strong corneal stroma.

Despite the evident allele dosage effect upon the expression of both *Col1a1* and *Col1a2* by keratocytes, with heterozygous cell lines expressing an intermediate amount relative to wild-type and homozygous lines, the corneas of heterozygous *Zfp469*^BCS^ mice are only slightly thinner than wild type. This mimics the observations from heterozygous carriers of LOF *ZNF469* and *PRDM5* mutations, who appear asymptomatic (Abu et al., 2008; Alazami et al., 2016; Aldahmesh et al., 2012; Avgitidou et al., 2015; Burkitt Wright et al., 2011; Christensen et al., 2010; Khan et al., 2012; Menzel-Severing et al., 2019; Micheal et al., 2016; Ramappa et al., 2014; Rohrbach et al., 2013; Skalicka et al., 2020) or show only mild corneal thinning and blue sclera (Burkitt Wright et al., 2011; Khan et al., 2010). This may reflect a critical threshold for the relative abundance of collagen type V to collagen type I in the cornea, which is known to have a major role in determining both the number of fibrils that form and determining their diameter (Dimasi et al., 2010; Sun et al., 2011; Wenstrup et al., 2004). Compared to skin, where collagen type V comprises only 2-5% of total fibrillar collagen, the relative ratio of collagen type V to collagen type I in the cornea is normally 5-10-fold higher. In our mouse model of BCS, this relative ratio is increased even further as the amount of collagen type I is reduced in both the corneal stroma and in skin. *ZNF469* mutations have been shown to disrupt deposition of collagen type I by dermal fibroblasts (Burkitt Wright et al., 2011), in agreement with our observation of decreased dermal thickness and collagen type I abundance in tail skin from *Zfp469*^BCS/BCS^ mice. However, this is the first report of mutation in Zfp469 disrupting the expression of collagen type I in the corneal stroma, and by primary keratocytes. Consistent with the role of collagen type V as a rate-limiting fibril nucleator, this appears to promote fibril nucleation from the depleted pool of collagen type I synthesised by keratocytes in *Zfp469*^BCS/BCS^ corneal stroma. This results in the extremely thin corneal stroma, comprising smaller diameter and more tightly packed collagen fibrils than in wild-type mice, which explains the phenotype observed in our model of the disease and in BCS patients.

Extreme thinning of the cornea was observed at 1 month of age in *Zfp469*^BCS/BCS^ mice, and BCS cases have been reported in humans soon after birth (Ramappa et al., 2014), suggesting that this disorder is initiated during development. This has also been observed in a mouse model for Marfan syndrome, another connective tissue disorder, which shows reduced CCT in *Fbn1^+/−^* mice from embryonic day (E)16.5 onwards (Feneck et al., 2020). The corneal stroma is established during early development, with collagen deposition by presumptive corneal stromal cells observed at E13 in the mouse (Feneck et al., 2019). As development proceeds, organised collagen fibrils begin to form, directed by keratocyte cell extensions into a parallel arrangement. Collagen type I appears to play a key role in this process, as complete knockout of *Col1a1* in the Mov13 mouse results in reduced corneal thickness at E16 and structural disorganisation of developing fibrils (Bard and Kratochwil, 1987). Temporal regulation of *Zfp469* expression in the mouse during embryonic corneal development was seen in the publicly available RNAseq dataset (NCBI GEO: GSE121044) comparing transcriptomes of periocular mesenchyme isolated at E10.5 with corneas isolated at E14.5 and E16.5 (Ma and Lwigale, 2019). This revealed that *Zfp469* is not expressed at E10.5 but is induced by E14.5, at which point neural crest-derived mesenchymal cells have migrated into the space between the surface ectoderm and the lens vesicle. *Zfp469* remains expressed at E16.5, by which point the presumptive stroma is well established. This expression pattern agrees with that reported for *ZNF469* during development of the cornea in the chick (Bi and Lwigale, 2019) and coincides with the induction of temporally regulated signalling pathways important for corneal development, and with increasingly high expression levels of *Col1a1*, *Col1a2* and other ECM components important in the stroma (Bard and Kratochwil, 1987; Ma and Lwigale, 2019). As the cornea matures, up to 8 weeks of age in the mouse, or 3 years of age in humans, collagen fibrils are further organised into additional layers of the established lamella. Our work suggests that the corneal thinning observed in *Zfp469*^BCS/BCS^ mice arises from a deficiency of collagen type I expression by keratocytes due to mutation in *Zfp469*. There does not appear to be a paucity of keratocytes in the stroma of mature cornea but we cannot exclude an impact on differentiation from neural crest cells or upon proliferation of keratocytes at early stages of development. Future work using our mouse and cellular models of disease will determine (1) whether corneal thinning in BCS is established during very early stages of development; (2) how ZNF469 regulates expression of collagen type I genes; and (3) whether there is a therapeutic window of opportunity for the modulation of ZNF469 function.

## MATERIALS AND METHODS

### Bioinformatics

A literature review was performed to identify mutations in ZNF469 that have been reported in BCS cases. Using the search term ‘ZNF469’, 12 case reports of ZNF469 mutations were identified, with a total of 30 different mutations occurring either as compound heterozygotes or homozygous in 53 cases of BCS. All mutations were mapped to transcript NM_001367624.2 and protein NP_001354553.1 using Ensembl Variant Effect Predictor (www.ensembl.org/Tools/VEP). The Genome Aggregation Database (gnomAD v3.1; https://gnomad.broadinstitute.org/) was searched for BCS mutations that may be present in the general population. Protein sequence alignments were performed using Clustal Omega (www.ebi.ac.uk/Tools/msa/clustalo/). Uniprot (www.uniprot.org/) and SMART (http://smart.embl-heidelberg.de/) were used to identify positions of C2H2 zinc-finger domains and compositional bias in the amino acid sequence.

### Preparation of reagents for genome editing

Paired sgRNA for use with nickase Cas9 were designed using the tool available at http://crispr.mit.edu (May 2017). Three pairs were designed and top and bottom strands ordered as 5′ phosphorylated single-stranded DNA oligos (Integrated DNA Technologies) with BbsI-cloning compatible ends for cloning into pX458 as described previously (Ran et al., 2013). pSpCas9(BB)-2A-GFP (pX458) was a gift from Feng Zhang (Addgene, 48138; RRID:Addgene_48138). sgRNA sequences are listed in Table S1. A ssODN repair template with 56 bp and 54 bp 5′ and 3′ homology arms, respectively, was designed to insert a V5 epitope tag and a premature stop codon into mouse Zfp469 at p.Gly634, and ordered as a PAGE Ultramer DNA Oligo (Integrated DNA Technologies). After verification of correct sequence by Sanger sequencing, plasmid DNA for each sgRNA was used to test the efficiency of on-target editing at the *Zfp469* locus in MEFs. After selection of the most efficient pair of sgRNAs, plasmid DNA was used as a template in PCR to amplify sgRNA 1 and 3 sequences using primers shown in Table S1. DNA was purified using Qiagen PCR purification columns, and 1 µg of purified PCR product was used as template for *in vitro* transcription using the HiScribe T7 High Yield RNA Synthesis Kit (New England Biolabs, E2040S) following the manufacturer's instructions.

### Testing sgRNA in mouse embryonic fibroblasts

Wild-type MEFs (200,000/reaction) were transfected with 2 µg pX458 plasmid containing sgRNA using Neon electroporation (1350 V, 30 ms and 1 Pulse). Transfected cells were incubated for 72 h in a humidified 37°C 5% CO_2_ incubator, after which cells were collected. The efficiency of sgRNA-mediated DNA cleavage at the on-target locus was determined using a GeneArt Genomic Cleavage Detection Kit (Invitrogen) following the manufacturer's instructions. Primers used for target amplicon amplification were as follows: forward, 5′-TTCATCTCTGTCACCGCCAT-3′, and reverse, 5′-GAAGGGGACAGTCTGGTTGT-3′.

### Genome editing in mice

Cytoplasmic injection of a 20 µl aliquot, containing 50 ng/µl SpCas9n mRNA, 50 ng/µl sgRNA and 150 ng/µl donor oligonucleotides into C57BL/6J mouse zygotes was performed, followed by immediate transfer to pseudopregnant CD1 females. Founder mice were genotyped by extracting DNA from earclips in DNAreleasy (Anachem) following the manufacturer's instructions. Primers (forward, 5′-TTCATCTCTGTCACCGCCAT-3′, and reverse, 5′-GAAGGGGACAGTCTGGTTGT-3′) were used to amplify DNA surrounding the editing target site from crude DNA by PCR using DreamTaq Green PCR Master Mix (Thermo Scientific). Sanger sequencing was used to verify editing and repair. One founder mouse with in-frame repair was identified and used to establish the *Zfp469*^BCS^ line by breeding with wild-type (WT, ^+/+^) C57BL/6J.

### Mouse husbandry

Experiments on mice were performed with UK Home Office project licence approval. Animals were housed in a facility on a 12-h light/dark cycle with unrestricted access to food and water. All mice were euthanised in accordance with UK Home Office guidelines. Heterozygous F1 offspring of the founder mouse were interbred to generate the subsequent generations of *Zfp469*^BCS^ mice used in this study, maintained on the C57BL/6J strain background. Litters were genotyped as for the founder mice, or outsourced to Transnetyx (Cordova, TN, USA) using allele-specific custom probes. Male and female mice were used in this study.

### Slit lamp examination

The anterior segment of the eyes of three *Zfp469*^+/+^, 3 *Zfp469^+/^*^BCS^ and 3 *Zfp469*^BCS/BCS^ mice were examined at 3 months of age using a slit lamp biomicroscope. Mice were examined without anaesthesia, and images were taken with a digital camera.

### Measurement of eye size

Freshly enucleated eyes from two homozygous mice and three wild-type mice were placed upon a flat surface, and measured from the front of the cornea to the posterior just left of the nerve four times using calipers. An average measurement was calculated for each eye.

### OCT

AS-OCT images were captured using a Heidelberg Spectralis OCT. Mice aged 1 month, 3 months and 6 months of age were anaesthetised by inhalation of isoflurane, and corneas were imaged using an anterior segment lens. To test corneal distortion after pupil dilation, a drop of 1% tropicamide and then a drop of 2.5% phenylephrine hydrochloride was added to each eye prior to AS-OCT imaging. Systane Ultra Lubricant (Boots) eye drops were used to prevent the eyes drying out.

Corneal dimensions, including central and peripheral corneal thickness and ACD, were determined from cross-sectional corneal images that passed through the centre of the pupil using ImageJ software (National Institutes of Health). CCT and PCT were determined by measuring the linear distance between the anterior and posterior corneal surfaces in the central cornea and peripheral corneal, respectively. ACD was the distance between the lens and the posterior surface of the central cornea. The measurements were obtained in pixels and the appropriate pixel to μm conversion factor was applied, relative to a 200 µm scale bar. One measurement was made for each scan of both eyes for each mouse. A minimum of three male and three female mice per genotype were used at each stage (1, 3 and 6 months of age).

### TEM

For TEM, corneas from three *Zfp469*^+/+^ and three *Zfp469*^BCS/BCS^ female mice aged 9 months were removed and fixed in 3% glutaraldehyde in 0.1 M sodium cacodylate buffer (pH 7.3) for 2 h, then washed in three 10-min changes of 0.1 M sodium cacodylate. Corneas were postfixed in 1% osmium tetroxide in 0.1 M sodium cacodylate for 45 min, and washed in three 10-min changes of 0.1 M sodium cacodylate buffer. These samples were dehydrated in 50%, 70%, 90% and 100% ethanol (3×) for 15 min each, then in two 10-min changes in propylene oxide. Samples were subsequently embedded in TAAB 812 resin. Sections (1 μm) were cut using a Leica Ultracut ultramicrotome, stained with Toluidine Blue and viewed in a light microscope to select suitable areas for investigation. Ultrathin sections (60 nm) were cut from selected areas, stained in uranyl acetate and lead citrate, and then viewed in a JEOL JEM-1400 Plus TEM. Representative images were collected using a GATAN OneView camera. Multiple high-magnification (35,000×) non-overlapping cross-sectional images were taken from the anterior stroma in the central cornea of each sample. Fibril dimensions were determined using ImageJ to analyse multiple images per sample across a defined region of interest in each image. Fibril diameter was measured by taking two measures perpendicular to each other and an average used for each fibril. Fibril density was calculated as the number of fibrils in the known area of the region of interest in each image. Microsoft Excel and GraphPad Prism were used for data analysis. Imaging and measurements were performed blinded to genotype.

### Histology

Mice were euthanised at the appropriate age by cervical dislocation before eyes were enucleated and tails were removed for fixing in 4% paraformaldehyde (PFA) solution overnight at 4°C. Following this, tails were washed three times with 6% EDTA for 1 week each. For wax preservation, tissue samples were removed from PFA or EDTA, and were subsequently dehydrated by successive washes in 70%, 80%, 90% and 100% ethanol, and xylene twice, and then embedded in paraffin for 45 min.

H&E and Masson's trichrome staining were performed on 8-μm paraffin-embedded tissue sections using standard procedures. Images were captured on a Zeiss Axioplan 2 brightfield microscope. Immunostaining was performed using 5-8-μm paraffin-embedded sections after antigen retrieval in citrate buffer (pH 6). Slides were rinsed with water, washed twice with Tris-HCl buffered saline with 0.1% Tween 20 (TBST) and then blocked in 4% heat-inactivated donkey serum in TBST for 30 min at room temperature. Primary antibodies (goat anti-type I collagen, 1310-01, Southern Biotech; and goat anti-type V collagen, 1350-01, Southern Biotech) were diluted 1:100 in 4% donkey serum in TBST and incubated overnight at 4°C. After three washes in TBST, Alexa Fluor secondary antibody diluted 1:250 in 4% donkey serum in TBST was added to the slides for 1.5 h at room temperature. Slides were washed three times in PBS prior to mounting coverslips with Prolong Gold Antifade Mountant with DAPI (Invitrogen). Slides were imaged on a Nikon Confocal A1R microscope for image processing using NIS-Elements or ImageJ.

### Corneal protein extraction

For proteomic analysis, six pooled corneas (from three different animals at 2 months of age) for each genotype were subject to mechanical grinding and five rounds of freeze-thawing in RIPA buffer [150 mM NaCl, 50 mM Tris (pH 8), 1% NP-40, 0.5% sodium deoxycholate and 0.1% SDS] plus cOmplete EDTA-free protease inhibitor cocktail (Roche). After centrifugation at 16,200 ***g*** for 10 min at 4°C, supernatants were recovered and protein concentration determined using a Bio-Rad protein assay according to the manufacturer's instructions, with bovine serum albumin as standard.

### Gel electrophoresis and western blotting

Samples containing equal amounts of total protein were mixed with 1× Reducing Agent (Invitrogen by Thermo Fisher Scientific) and 4× NuPAGE LDS (lithium dodecyl sulfate) Sample Buffer (Invitrogen by Thermo Fisher Scientific). Samples were denatured by heating to 80°C for 15 min. Gel electrophoresis was performed using 4–12% Bis-Tris NuPAGE gels (Invitrogen by Thermo Fisher Scientific) run in 1×MOPS (3-(N-morpholino)propanesulfonic acid (Invitrogen by Thermo Fisher Scientific). Proteins were transferred to Hybond-P PVDF membranes (GE Healthcare) in 1×NuPAGE Transfer Buffer (Invitrogen by Thermo Fisher Scientific) containing 20% methanol (Fisher). Membranes were blocked in 5% non-fat milk in PBS with Tween 20 [3.2 mM Na_2_HPO_4_, 0.5 mM KH_2_PO_4_, 1.3 mM KCl, 135 mM NaCl and 0.05% Tween 20 (pH 7.4)] before being incubated with primary antibodies (anti-telo-collagen type I, A1, ABT256, Sigma-Aldrich; anti-fibronectin-1, F3648, Sigma-Aldrich) followed by anti-rabbit IgG horseradish peroxidase-conjugated secondary antibody. Protein bands were visualised using enhanced chemiluminescence western blotting detection reagents (GE Healthcare) and by exposure of the membrane to Kodak BioMax XAR film (Sigma-Aldrich). Films were developed using a phosphorimager X-ray machine (Konica Minolta). Densitometric analysis of scanned films was performed using ImageStudioLite software (Li-Cor).

### Mouse embryonic fibroblast generation and culture

MEFs were isolated from E13.5 embryos from heterozygous *Zfp469^+/^*^BCS^ crosses. Heads and organs were removed from embryos and the remaining sample was digested with trypsin for 10 min at 37°C to generate a cell suspension. Cells were pelleted by centrifugation at 253 ***g*** for 4 min and then cultured in Opti-MEM plus 10% fetal calf serum (FCS) plus pencillin/streptomycin. MEFs were used between passages three and six.

### Mouse primary keratocyte generation and culture

Mouse corneal stroma keratocytes were isolated as described previously (Zhang et al., 2016), with slight modification. In brief, pups were euthanised at P2, after which eyes were dissected, rinsed in PBS and corneas cut out along the scleral rim. Two corneas (per mouse) were incubated in 15 mg/ml Dispase (4942078001, Roche) in Dulbecco's modified Eagle medium (DMEM; Gibco) plus penicillin/streptomycin for 30 min at room temperature. Epithelial cells were detached by gentle shaking and the corneas rinsed in PBS, before incubation in digestion buffer containing 2 mg/ml collagenase (Gibco) and 0.5 mg/ml hyaluronidase (Sigma-Aldrich) in DMEM at 37°C for 30 min, with vortexing every 5 min. Samples were centrifuged at 300 ***g*** for 5 min before the supernatant was removed, and trypsin/versine was added for a further incubation at 37°C for 10 min. Cells were recovered by centrifugation at 300 ***g*** for 5 min, the supernatant discarded and cells resuspended in culture medium (DMEM, 10% FCS, 1% pencillin/streptomycin) for plating. Primary keratocytes (corneal stromal fibroblasts) were maintained in a humidified 37°C 5% CO_2_ incubator, subcultured at 80% confluency and were used between passages three and six.

### Isolation of RNA, cDNA synthesis and real-time qPCR

RNA was isolated from corneal samples dissected from four wild-type, two heterozygous and four homozygous mice aged 3 months, and the corneas processed as described previously to obtain cells for primary keratocytes culture. After treatment with trypsin/versene, the resulting cell pellet was resuspended in Buffer RLT and total RNA was prepared using an RNEasy Kit (Qiagen). RNA was also purified from primary mouse keratocytes isolated from P2 pups at passage four using the RNEasy Kit. Reverse transcription was performed using a Transcriptor High Fidelity cDNA Synthesis Kit (Roche) and random hexamer primer, in accordance with the manufacturer's instructions. qPCR was performed using Taqman Gene Expression assays (*Hprt*, Mm01324427_M1, *Col1a1*, Mm00801666_g1, *Col1a2*, Mm00483888_m1, Col5a1, Mm00489229_m1; *Kera*, Mm00515230; *Fn1*, Mm01256744_m1; *Prdm5*, Mm00510567_m1; *Gapdh*, Mm99999915_g1; custom assays targeting the start of the *Zfp469* transcript and the V5 insertion; Applied Biosystems) with Taqman Universal MasterMix II, no UNG (Applied Biosystems). Assays were run on an ABI PRISM 7900 thermocycler with each sample in triplicate. *Hprt* and *Gapdh* were used as housekeeping genes. Data were analysed using the 2^−ΔΔCT^ method for relative quantitation.

### Proliferation assays

Proliferation assays were performed using 50,000 primary mouse keratocytes or MEFs from four independent cell lines for each genotype at passage three. After 96 h in complete culture medium the cells were trypsinised and counted using a haemocytometer. The number of population doublings in 96 h was used to calculate the doubling time for each cell line in hours.

### Adhesion assay

The adhesion of primary mouse keratocytes to the ECM components collagen type I, collagen type IV, fibrinogen, fibronectin and laminin was quantitatively tested using a CytoSelect Adhesion Assay Kit (Cell Biolabs, San Diego, CA, USA) in accordance with the manufacturer's instructions. In brief, three wild-type lines, obtained from three different mice, were compared to three homozygous mutant lines, also obtained from three different mice. A total of 75,000 cells in serum-free DMEM were seeded into the wells of a 48-well plate precoated with the ECM components, and then incubated for 45 min at 37°C. The medium was removed and the plate washed five times with PBS to remove non-adherent cells before the addition of cell stain solution to each well. After incubation for 10 min at room temperature, wells were washed five times with PBS and allowed to air dry. Extraction Solution was added and after 10 min incubation with shaking, 150 µl of each sample was used to measure absorbance at 560 nm in a TECAN M200 Pro plate reader (Tecan, Switzerland).

### Pro-collagen 1A1 ELISA

Primary mouse keratocytes were seeded at 75,000 cells per 10-cm plate and grown to confluency in medium containing FCS. Cells were washed in PBS, and the medium was changed to serum-free for a further 7 days. Cell culture supernatants were collected and assayed for the secretion of pro-Col1A1 using a Human Pro-Collagen I alpha 1 DuoSet ELISA Kit (DY6220-05, R&D Systems) in accordance with the manufacturer's instructions.

### Generation of cell-derived matrices

Primary mouse keratocyte cells were used to generate CDMs following a published protocol (Kaukonen et al., 2017). In brief, multi-well tissue culture plates and glass coverslips were coated with sterile 0.2% (w/v) gelatin diluted in PBS for 1 h at 37°C. Coverslips were washed with PBS and then crosslinked with 1% (v/v) glutaraldehyde for 30 min at room temperature, prior to quenching with sterile 1 M glycine for 20 min. Coverslips and plates were then washed with PBS and incubated in DMEM, 10% FCS and 1% penicillin/streptomycin for 1 h before use.

Primary mouse keratocytes were seeded 2×10^5^ cells/well in six-well plates. Cells were cultured in DMEM, 10% FCS and 1% penicillin/streptomycin in a humidified 37°C 5% CO_2_ incubator until confluent. Medium was then supplemented with 50 μg/ml ascorbic acid. The medium was changed every 2 days for 20 days. After washing in PBS, cells were denuded by adding extraction solution (20 mM NH_4_OH and 0.5% Triton X-100 in PBS) to lyse the cells. Following two washes in PBS, DNA was digested with 10 μg/ml DNase I (Roche) in PBS for 30 min at 37°C, before washing twice more. CDMs in tissue culture plates were scraped into 2× LDS buffer with reducing agent added before denaturing at 90°C for 20 min. CDMs on coverslips were fixed in 10% formalin for 15 min at room temperature, washed with PBS and blocked in 30% donkey serum in PBS. Primary antibodies (1:250 anti-fibronectin-1, F3648, Sigma-Aldrich; 1:100 goat anti-type I collagen, 1310-01, Southern Biotech; and 1:100 goat anti-type V collagen, 1350-01, Southern Biotech) were diluted in 30% donkey serum in PBS and incubated on CDMs for 1 h at room temperature. After three washes in PBS, Alexa Fluor-conjugated secondary antibodies (donkey anti-goat IgG Alexa Fluor 488, A-11055, Thermo Fisher Scientific; and donkey anti-rabbit IgG Alexa Fluor 594, A21207, Thermo Fisher Scientific) were added for 1 h at room temperature. Finally, CDMs were washed three times in PBS and once in water before coverslips were mounted on slides using Vectashield Antifade mounting medium (Vector Laboratories). Imaging was performed using a Nikon Confocal A1R microscope. NIS-Elements or ImageJ was used for image processing and analysis.

### Statistics

Statistically significant differences between experiments were determined using an unpaired two-tailed Student's *t*-test, one-way ANOVA or Welch's ANOVA test in which variances between groups differed significantly. Post-hoc analysis using Tukey's or Dunnett's multiple comparisons tests was performed (GraphPad Prism v9.1.0). *P*<0.05 was considered significant. Experiments were performed a minimum of three times, unless indicated otherwise, and data are presented as mean±s.e.m. or mean±s.d.

## Supplementary Material

Supplementary information
