# Supplementary material for: A mouse model of brittle cornea syndrome caused by mutation in Zfp469
Source: Dis Model Mech. 2021 Sep 22;14(9):dmm049175. doi: 10.1242/dmm.049175 (PMC8476817; doi:10.1242/dmm.049175)
Supplement: Supplementary information [file dmm-14-049175-s1.pdf]

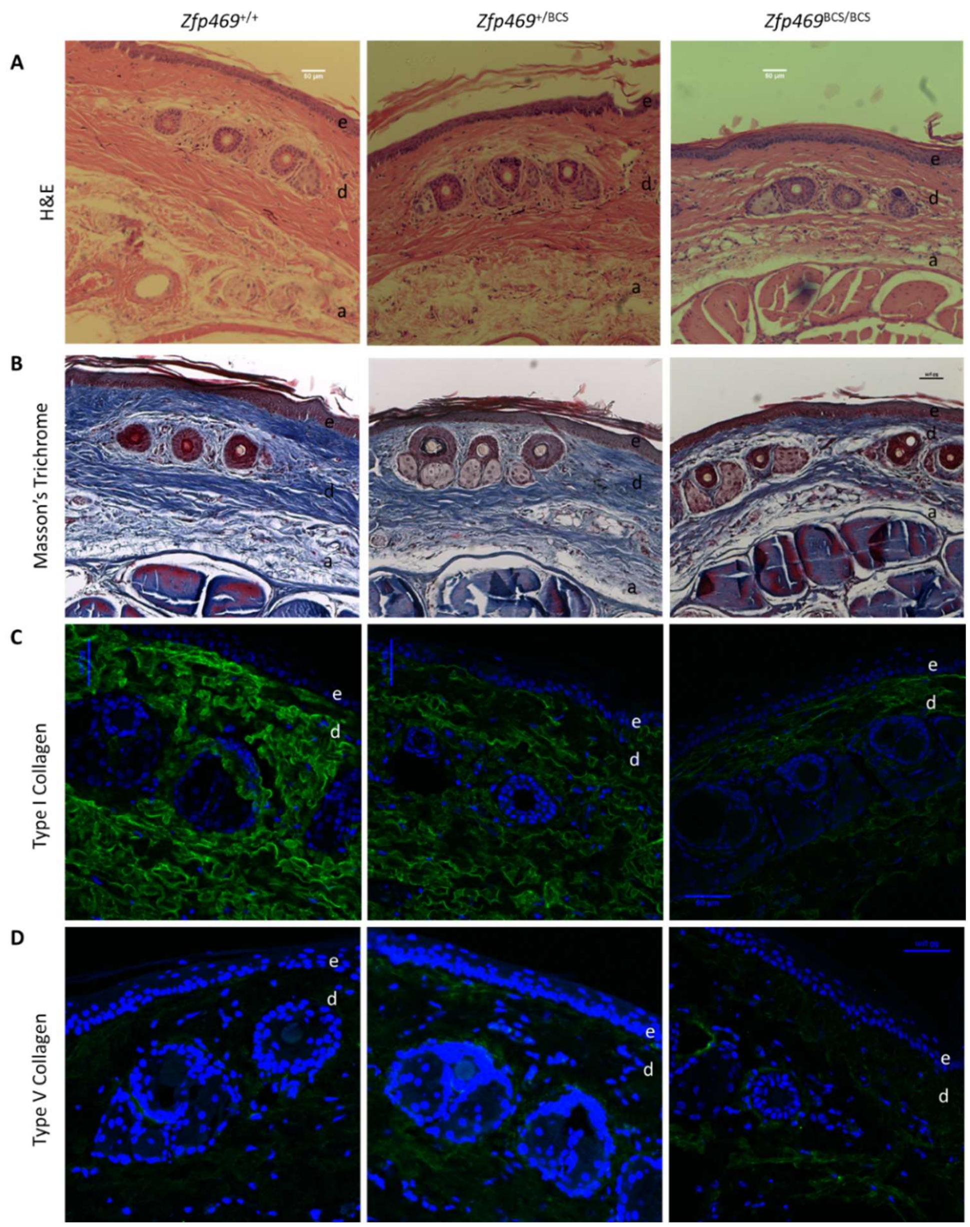

Fig. S1. Decreased dermal thickness and type I collagen abundance in *Zfp469*$^{BCS/BCS}$ tail skin. (A) Representative Hematoxylin and eosin (H&E) and (B) Masson's trichrome staining of sections of tail skin from *Zfp469*$^{+/+}$, *Zfp469*$^{+/BCS}$ and *Zfp469*$^{BCS/BCS}$ littermate male mice at 6 months of age shows thinning of the dermis (d) and subcutaneous adipose (a) layer in homozygotes (n=3 for each genotype). The collagen enriched dermal matrix is stained blue by Masson's Trichome stain, the epidermis (e) is stained red. Scale bars represent 50 µm. (C) Representative immunofluorescence images of tail skin sections stained for type I collagen (green) and (D) type V collagen (green) show decreased type I collagen staining in the dermis (d) of *Zfp469*$^{BCS/BCS}$ tail sections, but type V collagen staining is similar to that observed in wildtype tail sections (n=3 for each genotype). Nuclei are stained with DAPI (blue). Scale bars represent 50 µm.

Table S1. CRISPR-Cas9n genome editing sgRNA and repair template sequences and primers used for in vitro transcription. Guide RNA sequences shown in bold were used in CRISPR-Cas9 genome editing to generate the *Zfp469*^{BCS} line.

**Name**	**Sequence 5' - 3'**
gRNA1	TTGAAGGCATCCTCAGCCCCTGG
gRNA2	CTCCCACAAGAGCCCCTCACTGG
**gRNA1**	**TTGAAGGCATCCTCAGCCCCTGG**
**gRNA3**	**TCCCACAAGAGCCCCTCACTGGG**
gRNA4	AGGGAAGGCTTTGGCTGTCTCGG
gRNA5	TGAGGATGCCTTCAAGAGCCAGG
Repair template	CCACACGCTATCAATCCGAGACAGCCAAAGCCTTCCCTCTCCCCACAGAGGGACCA GGCAAACCGATTCCGAACCCGCTGCTGGGCCTGGATAGCACCGGCAAACCGATTCC GAACCCGCTGCTGGGCCTGGATAGCACCTGAGtGCAGAGACGGGTTGAAGGGCTT TCCTCCAGAGCCACCACCTCCACCGCCACC
mZNF469g1 T7 primer	TGTAATACGACTCACTATAGGttgaaggcatcctcagcccc
mZNF469g3 T7 primer	TGTAATACGACTCACTATAGGtcccacaagagcccctcact
Universal reverse	AAAAGCACCGACTCGGTGCC

Table S2. Loss of function mutation of Zfp469 is transmitted in the expected ratio in litters born from heterozygote x heterozygote crosses.

	**+/+ (N)**	**+/BCS (N)**	**BCS/BCS (N)**	**Total (N)**
**Male**	8	13	5	26
**Female**	11	23	10	44
**Obs**	19	36	15	70
**Exp**	17.5	35	17.5	

Genotypes of offspring obtained in 10 litters from heterozygote x heterozygote crosses for the *Zfp469*^{BCS} line were subject to Chi2 test, with no significant difference in genotype frequency between observed and expected numbers (N) (two-tailed p value = 0.3787).
